# Epigenomic Views of Innate Lymphoid Cells

**DOI:** 10.3389/fimmu.2017.01579

**Published:** 2017-11-13

**Authors:** Giuseppe Sciumè, Han-Yu Shih, Yohei Mikami, John J. O’Shea

**Affiliations:** ^1^Department of Molecular Medicine, Sapienza University of Rome, Laboratory Affiliated to Istituto Pasteur Italia – Fondazione Cenci Bolognetti, Rome, Italy; ^2^Lymphocyte and Cell Biology Section, Molecular Immunology and Inflammation Branch, National Institute of Arthritis and Musculoskeletal and Skin Diseases, NIH, Bethesda, MD, United States

**Keywords:** innate lymphoid cells, NK cells, epigenetic, regulomes, DNA accessibility, transcriptomes, transcription factors

## Abstract

The discovery of innate lymphoid cells (ILCs) with selective production of cytokines typically attributed to subsets of T helper cells forces immunologists to reassess the mechanisms by which selective effector functions arise. The parallelism between ILCs and T cells extends beyond these two cell types and comprises other innate-like T lymphocytes. Beyond the recognition of specialized effector functionalities in diverse lymphocytes, features typical of T cells, such as plasticity and memory, are also relevant for innate lymphocytes. Herein, we review what we have learned in terms of the molecular mechanisms underlying these shared functions, focusing on insights provided by next generation sequencing technologies. We review data on the role of lineage-defining- and signal-dependent transcription factors (TFs). ILC regulomes emerge developmentally whereas the much of the open chromatin regions of T cells are generated acutely, in an activation-dependent manner. And yet, these regions of open chromatin in T cells and ILCs have remarkable overlaps, suggesting that though accessibility is acquired by distinct modes, the end result is that convergent signaling pathways may be involved. Although much is left to be learned, substantial progress has been made in understanding how TFs and epigenomic status contribute to ILC biology in terms of differentiation, specification, and plasticity.

## Introduction

The immune system employs a variety of effector cells that ensure protection against diverse types of infections. An important strategy for host defense is that distinct immune responses are evoked by different pathogens, one aspect being the production of selective cytokines (1, 2). Intracellular bacteria and viruses are usually eliminated through the so-called type 1 response, which is dominated by the release of interferon (IFN)-γ as a signature cytokine. Moreover, cells infected by these pathogens are recognized by immune cells with the ability to directly kill their targets by releasing perforin and granzymes and inducing programmed cell death (3–5). In contrast, parasites and worms evoke a type 2 response, characterized by the production of interleukin (IL)-4, IL-5, and IL-13, necessary to drive their elimination/expulsion (6, 7). Finally, extracellular pathogens and fungi are associated with production of the signature cytokines IL-17 and IL-22, which provide host defense at mucosal surfaces (8).

**Figure 1:**
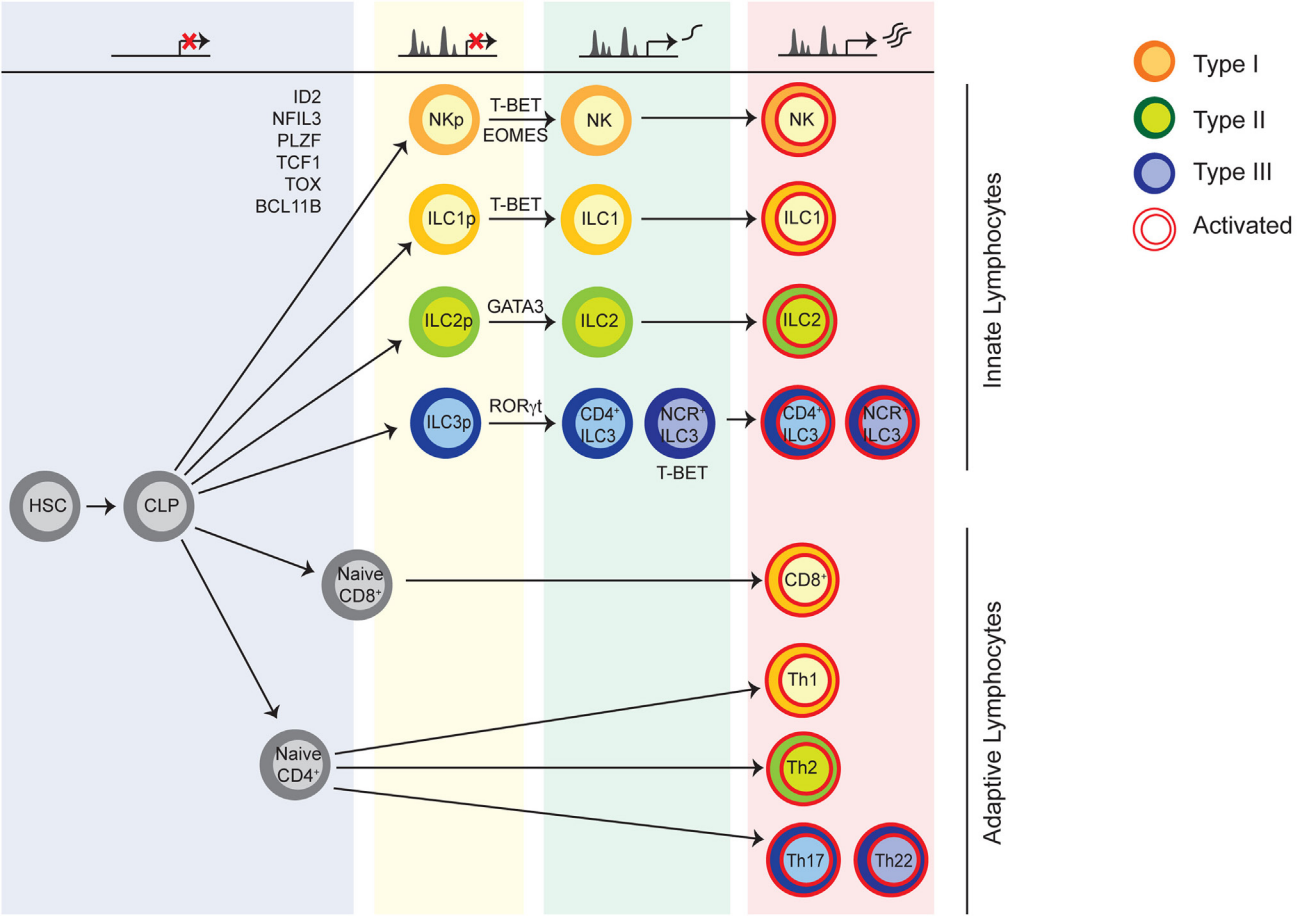
Innate lymphoid cells (ILCs) are divided into three groups analogously to T cell subsets. NK cells and other interferon-γ producing cells, namely ILC1, belong to the group of type 1 ILCs. Helper ILCs producing T helper (Th)2- and Th17/22-cytokines are termed ILC2 and ILC3, respectively. Type 3 ILCs comprise lymphoid tissue inducer-like cells and NCR^+^ ILC3. The different ILC subsets acquire their specific epigenomic features in a step-wise manner during development. In the course of specification, ILC precursors acquire regulatory elements for signature genes expressed by mature cells, while opposing fates are antagonized. During infection, ILCs and effector T cells undergo convergent epigenetic regulation. Transcription factors required during development and lineage-defining transcription factors for prototypical ILC subsets are shown.

In many respects, the effector functions described above have been mainly associated with the adaptive arm of the immune system, with a largely T cell-centric point of view (9). Although NK cells were recognized 40 years ago, other innate lymphocytes with helper features were unknown until recently (10). Based on the analogy with the effector functions of T cell subsets, innate lymphoid cells (ILCs) are currently divided into three groups (Figure 1) (11). Type 1 ILCs include both NK cells and ILC1 which may be viewed as the innate counterpart of CD8^+^ cytotoxic T lymphocytes, and T helper (Th) 1 cells, respectively. Similarly, ILC2 and ILC3 comprise helper lymphocytes that promote Th2- and Th17/22-related responses (12–16). Lymphoid tissue inducer (LTi)-like cells are included in the ILC3 group, as well as, ILCs expressing natural cytotoxicity receptors (NCRs), found in the mucosal tissues (17–22). More recently, the identification of an ILC subset that produces IL-10 and transforming growth factor (TGF)-β, and suppresses effector functions of other ILCs has broadened this view, adding yet another ILC subset that shares functional properties with regulatory T cells (23). Beyond these functions, several other concepts of T cell biology have been applied to ILCs, including memory-like responses of NK cells following viral infection (24–26) and the plasticity of ILCs that occurs in response to environmental changes (27, 28). Despite the similarities, an important distinction between innate and adaptive lymphocytes is the characteristic poised activation state of ILCs, as well as, their lack of antigen receptor engagement for acquisition of effector functions (29–31).

The field of ILC biology is an area of intense investigation and it is impossible to do justice to all the rapid advances. Not only are ILCs being considered in terms of host defense and immune mediated disease, but are emerging as players in the regulation of metabolic homeostasis, deposition of adipose tissue and, obesity, in both physiological and pathological conditions (32–35). However, in this short essay, we will focus on how the progress of next generation sequencing (NGS) technologies has provided new insights into some of the aspects of ILC biology from a genome-wide perspective. Clues to the nature of ILC identity and function are revealed by their global epigenomic features and transcriptomic programs, providing insights into heterogeneity and plasticity, and new paradigms for their relationships to T cells. These concepts can be integrated in the context of transcription factor (TF) networks, differentiation, microenvironment, and infections.

## Transcriptional Programs Defining ILCs

The transcriptional programs defining distinct ILC lineages have been investigated by using both genomic and genetic tools. These approaches have helped to elucidate shared and distinctive features of innate and adaptive lymphocytes, and the TFs required in regulation of ILC differentiation/functions. Although in homeostatic conditions ILCs share more genes with each other than they do with T cells (36, 37), the similarity between T cells and ILCs is exemplified by the common expression of a high number of genes and the clear reliance on many of the same lineage-defining transcription factors (LDTFs), also referred to as “master regulators” of T cell fate.

The expression of two LDTFs belonging to the T-box family, EOMES and T-BET (encoded by *TBX21*), is critical for NK cells. Deletion of *Eomes* in mouse leads to the loss of NK cells, which is not rescued by the expression of T-BET (38). On the other hand, *Tbx21^−/−^* NK cells show defects in cell turnover, trafficking, and functional properties (39). The constitutive expression of these two TFs helps to explain the poised features of NK cells and highlights functionalities shared with CD8^+^ T cells, although the latter upregulate T-BET and EOMES expression after activation. Transcriptomic analyses have shown that the poised state of NK cells is not restricted to the expression of *Ifng* and genes required for the cytotoxic machinery, but comprises multiple effector molecules transcribed in resting mouse NK cells and also in activated/effector CD8^+^ T cells (37).

In contrast to NK cells, ILC1 do not express EOMES and, instead, like Th1, require only T-BET for their development, as shown by *Tbx21^−/−^* mice (38, 40, 41). However, the ectopic expression of EOMES in ILC1 pushes their differentiation toward mature NK cells, suggesting that ILC1/NK conversion could involve induction of EOMES (42). Recently, cells with mixed ILC1/NK phenotype have been identified in mouse salivary gland, as well as, NK cells expressing EOMES and low levels of T-BET in human liver (43–45). Based on expression of cytokine/chemokine receptors and other surface markers, liver-resident ILC1 can be viewed as being related to NKT cells. More broadly though, the liver ILC1 program has greater global similarity to NK cells versus NKT cells (46). Although liver ILC1 are not considered prototypical cytotoxic ILCs, they do express high levels of the transcripts encoding for granzyme A and C (*Gzma, Gzmc*) and can kill through the expression of tumor necrosis factor-related apoptosis-inducing ligand (36, 46, 47). Together, these findings blur the boundaries among the different type 1 ILCs; although, strong evidence for functional sub-specialization of this group of cells is still limited.

Type 2 ILCs are dependent upon GATA-3 for their development, as this LDTF is for Th2 cells. Likewise, ILC3 require RORγt (encoded by *RORC*), which also promotes the fate of Th17/22 cells (12). Further complexity in the ILC3 group is provided by the combinatorial expression and requirement of T-BET, and GATA-3 in NCR^+^ ILC3 (40, 48–53). The evidence for expression of more than one LDTF in ILCs highlights the functional importance of a coordinated network of TFs in regulation of ILC transcriptional programs. However, ILCs are not alone in this regard—both adaptive and innate T cells are now appreciated to rely on a network of TFs working in conjunction, rather than on a single TF as previously proposed in the monolithic view of T cell development (9, 31, 54).

Together with the functional options acquired by “default” during development, the phenotype of mature ILCs can be skewed toward different fates through distinct environmental stimuli (51, 55–60). Cytokines previously linked to the processes of differentiation and plasticity of Th cells are now considered major drivers of the functional plasticity of ILCs. For instance, type 1 features in ILC2 can be induced by IL-12 (57, 58, 60), whereas ILC3/ILC1 transitions can be driven by IL-12 and IL-23 (51, 55, 56). In addition, both canonical and non-canonical pathways downstream of TGF-β signaling regulate NK/ILC1 conversion, along with imprinting of the ILC1 features, NK cell activity, and differentiation (43, 61–65). Thus, the environment—including tissue-specific signals—becomes a fundamental element to consider in the regulation of global gene expression in ILCs, which can modify the effects of lineage identity driven by LDTFs (36, 66–68). In this regard, the role for signal-dependent TFs, such as AHRs, RORs, NOTCHs (including the induction of downstream TFs, BCL11B, and GFI1), and STATs has become evident in several settings (29, 69–73).

Our understanding of the functions of key TFs involved in ILC differentiation is often limited by the total loss of cells observed in knockout mice, which limits the ability to identify direct targets. To overcome this issue, deletion of a single TF allele may still permit cell development and thus, permit evaluation of the consequence of reduced TF expression. This genetic approach, combined with transcriptomic analysis, has been useful to identify a discrete number of genes directly regulated by T-BET and, more recently, STAT5 in ILCs (53, 74). In the case of STAT5, the presence of two distinct genes encoding *Stat5a* and *Stat5b*, provides multiple genotypes, and ILC phenotypes, to explore. Deletion of the entire *Stat5a/b* locus has profound effects on lymphoid development and NK cells were absent in the few mice that survived this genetic lesion (75). More recently, lack of NK cells has been observed in mice carrying a deletion of the *Stat5a/b* locus specifically in cells expressing NKp46 (76). The selective ablation of only one gene (using *Stat5a^−/−^* or *Stat5b^−/−^* mice) highlighted a major role for STAT5B upon STAT5A, in the maintenance and *in vitro* proliferation of NK cells (77). Thus, the employment of mice carrying only one allele for *Stat5a* (*Stat5a^+/−^Stat5b^−/−^*) or *Stat5b* (*Stat5a^−/−^Stat5b^+/−^*) represented a suitable compromise allowing to interfere with the amount of STAT5 without reaching a total ablation (74). As well, RNA-seq and ChIP-seq analyses helped to reveal a direct and constitutive role for STAT5 in maintaining the expression of genes defining the identity of NK cells, beyond its known role in regulation of survival and proliferation. Moreover, reduction in STAT5 signaling perturbed other NCR^+^ ILCs, including ILC1 and NCR^+^ ILC3, while having less of an impact on ILC2 and LTi-like ILC3. This preferential, or hierarchical, requirement for STAT5 is explained in part by a direct role in regulation of T-BET expression on NCR^+^ ILCs.

Amidst this complexity, what is clear is that there seems to be no single “master regulator” for ILCs or ILC subsets (akin to *MyoD* in muscle or *Pax5* in B cells); instead, as with T cells, there appears to be complex orchestration of TFs that presumably exert their effect in a combinatorial manner and LDTFs act in concert with SDTFs (78).

## Regulomes

The hard-wired effector functions of ILCs have been appreciated since the observation of the constitutive transcription of the *Ifng* gene in resting NK cells, favored by an accessible chromatin conformation of its promoter (79, 80). Thousands of accessible regions have been defined, which spread throughout the chromatin allowing/restraining access to TFs and other transcriptional regulators and determining the final outcome of gene expression. These sites include not only promoters, but also non-coding regulatory elements (REs), such as enhancers, silencers, repressors, and insulators, and are called, overall, regulomes (81). The different types of REs can be discriminated by the presence of selective histone modifications or histone modifiers. For instance, trimethylation of histone H3 at lysine 4 (H3K4me3) is a histone mark enriched at the promoter of active genes; while H3K4me1, H3K4me2, acetylation of H3K27 (H3K27ac), and the presence of the acetyltransferase p300 are found at enhancer sites (82). Below, we will discuss how the ILC epigenomic programs contribute to ontogeny and function.

### Ontogeny of ILCs

In sharp contrast to T cells, signals from antigen receptors are not required for ILC effector function nor development (29–31). Instead, multipotent ILC precursors, including the α-lymphoid progenitor, early innate lymphoid progenitor, common helper innate lymphoid progenitor, and ILC progenitor, are regulated in mouse by the programmed expression of several TFs, including ID2 (inhibitor of DNA binding-2), TCF1 (encoded by *TCF7*), PLZF (encoded by *ZBTB16*), TOX, and NFIL3 (27, 83, 84). The above-mentioned ILC precursors progressively lose their multipotentiality, becoming unipotent ILC precursors, and a new set of TFs is required before lineage diversification, such as BCL11B for ILC2 or RUNX3 for ILC1 and ILC3 (85–88). However, expression of these TFs is not limited to the early stages of differentiation, as in the case of the basic helix-loop-helix TF, ID2, which is both required for the commitment of the entire ILC lineage and homeostasis of mature cells. One of ID2’s primary roles is to inhibit the functions of E proteins, blocking T and B cell development in favor of the ILC fate (89–94). Unlike PLZF which is required for both invariant NKT (iNKT) cell and ILC development, deficiency of *Id2* does not alter the development of iNKT cells, indicating both specific and overlapping requirements for innate and innate-like T cell ontogeny (41, 94, 95). The early steps of ILC differentiation are also characterized by the requirement of the basic leucine zipper TF, NFIL3 (96–98). In *Nfil3^−/−^* mice, generation of B, T, and NKT cells is not affected, while development of NK cells (99–102) and other ILC subsets (35, 61, 98, 103) is highly impacted. However, in the context of mouse cytomegalovirus infection, the signals provided by the triggering of the activating NK cell receptor Ly49H and by the proinflammatory cytokine IL-12 can overcome the requirement for this TF, leading to generation of NK cells with intact functional properties and ability to mediate memory responses (104). The source of these NK cells remains unknown, but their origin could be explained by the presence of NFIL3-alternative pathways of NK cell generation, as shown for other type 1 ILCs, such as salivary gland NK cells (105, 106) and liver ILC1 (107), which can develop in the absence of NFIL3. Although during lymphoid development, the range of NFIL3 action is restricted to ILCs, this TF, as well as ID2, can have a broader role, being required for optimal production of IL-13 and IL-10 by adaptive and innate T cells and for terminal differentiation of Th17 (108–110).

How the expression of these and the other TFs expressed during ILC development impacts the acquisition of lineage-specific REs has not been investigated; although at present, the small numbers of such cells represent a technical challenge. However, when ILC precursors become unipotent, progressively acquire distinctive lineage-specific REs (66). Indeed, after lineage specification committed NK and ILC2 precursors show features of chromatin accessibility typically found in fully developed NK cells and ILC2, including not only loci related to signature cytokines but also genes encoding for molecules acquired only at late stages of differentiation, as is the case of KLRG1 (66). Another important aspect is that chromatin accessibility of lineage-specific genes starts to diverge at the precursor stage. Consequently, the loci encoding type 2 cytokines are not accessible in NK precursors, whereas the *Ifng* locus is not accessible in ILC2s, indicating that formation of the signature features of chromatin accessibility occurs during development, defining their effector function as well as restraining their alternative fates. In this regard, the lysine methyltransferase G9a, that catalyzes the repressive histone mark H3K9me2 (dimethylation of histone H3 at lysine 9), plays a key role in preserving the ILC2 fate (111). When its deletion is applied to the entire mouse hematopoietic system, both development in the bone marrow and homeostasis of tissue-resident ILC2 are highly impacted. Evidence of the repression of alternative fates mediated by G9a is the increased expression of genes associated to the type 3 response occurring after its deletion (111). Recently, a human ILC precursor able to give rise to all known ILC subsets has been defined in the peripheral blood (112). This precursor expresses several TFs related to murine ILC development, such as ID2, GATA-3, TOX, and TCF7, and its identity has been defined through the analysis of the genome-wide distribution of the histone modification H3K4me2 (dimethylation of Histone H3 at lysine 4) (112). This histone modification is present on both active and poised loci (113). Although LDTFs, as EOMES, TBX21, RORC, and other molecules present on mature ILCs are not expressed (including IL23R, CCR6, and signature cytokines), these genes show the presence of H3K4me2, indicating that they are epigenetically poised (112). Thus, both in human and mouse, ILC signature genes can acquire a complete set of REs at the precursor level, while the expression of these genes is induced/upregulated only after terminal differentiation.

### Lineage Relationships and Identity

The elucidation of both mouse and human ILC regulomes has provided global molecular evidence for their poised state and also for the current functional classification. Since ILC nomenclature is based on the common features with T cells, a compelling question is the extent to which ILCs and T cells are appropriately viewed as two distinct “lineages” or whether their epigenomic features converge reflecting their effector features. Interestingly, in homeostatic conditions, the chromatin accessibility of distinct ILC and T cell subsets seems to be divergent, as observed in both human and mouse (66, 67). Similar conclusions have been obtained through analyses of a class of enhancers highly linked with cell identity, called stretched- or super-enhancers (SE) (67). In contrast, the patterns of chromatin accessibility of ILCs and T cells converge in the infection settings, as a part of the shared transcriptional/epigenetic programs. Indeed, upon *Nippostrongilus brasiliensis* infection in mouse, the transition from naïve to Th2 cells requires a wholesale remodeling of the chromatin but nonetheless, Th2 regulomes converge with those of ILC2 (66). In the same way, after human cytomegalovirus infection, the DNA-methylation patterns of adaptive NK cells parallel those of cytotoxic/effector CD8^+^ T cells (114). These NK cells show downregulation of the TF PLZF and peculiar functional properties which occur thanks to the regulation of the DNA-methylation state of key NK cell genes, including TFs and signaling proteins (114, 115).

When compared with transcriptomes, ILC regulomes are reportedly less dependent on acute environmental effects, and more sensitive to discriminate lineage identity (66); however, the extent to which ILC regulomes are malleable is clearly worthy of further investigation. Of note, after microbiota depletion, both intestinal ILC1 and ILC2 lose a portion of their signature REs and acquire type 3 features; while their global identity is preserved (68). Definition of ILC identity relies on the presence of very well-defined clusters of enhancers, as shown by the genome-wide distribution of p300 and histone marks (66–68). In contrast, our understanding of the specific role for single/specific REs in regulation of gene expression in ILCs is largely unknown. Another area that is in its infancy is the understanding of the function of cis-REs associated with expression of long non-coding RNAs (lncRNAs). One lncRNA termed RNA-demarcated regulatory region of ID2 (*Roid*) is essential for the expression of ID2 selectively on mature type 1 ILCs, in mouse (116). Beyond the initial role in driving differentiation of the ILC lineage, ID2 is required for homeostatic regulation of differentiated cells, such as type 1 and type 3 ILCs (116, 117), while the ectopic expression of ID1 in the thymus induces a general increase of the numbers of ILC2 in mice (118). Through regulation of the accessibility of the *Id2* locus, this cis-RE and its related lncRNA, *Roid*, control identity, differentiation, and functions of both NK and ILC1. These data point out that, along with TFs and REs, lncRNAs can represent another layer of epigenetic regulation in ILCs (119). In this context, another lncRNA, lncKdm2b, contributes to the maintenance of murine ILC3 by promoting the expression of ZFP292, recruiting SATB1, and the nuclear remodeling factor complex to the *Zfp292* promoter (120).

## Recent Advances from Single-Cell Analyses

The development of new strategies for single-cell analysis has made possible the characterization of the expression profiles of transcripts and proteins (121). Gene expression at the single-cell level can now be profiled by several single-cell RNA-sequencing (scRNA-seq) protocols, with different sensitivity, accuracy, cost-efficiency, and drawbacks (122, 123). Technical challenges notwithstanding, single-cell resolution has revealed substantial heterogeneity among ILCs. In mouse, 15 transcriptional states have been identified for intestinal CD127^+^ ILCs, including two transcriptional profiles that did not fall in any previously recognized ILC category (68). Similarly, the features of CD127^+^ ILCs and NK cells isolated from tonsils have been dissected in humans (124). The scRNA-seq approach has been also a key to clarify the heterogeneity of ILC precursors in mouse, which has led to establish the relevance of BCL11B in the differentiation process and to the identification of PD-1 as an early checkpoint in ILC2 development (125). Notably, treatment with PD-1 antibodies acts on ILCs in mouse models of infection (influenza and *N. brasiliensis*) and papain-induced acute lung inflammation (125, 126). In the context of fetal development, the requirement of NOTCH in lymphoid progenitors has been also dissected, such as the lineage relationship between LTi cells and other ILC lineages. These findings suggest a clear bifurcation in fetal lymphoid progenitors which involves an LTi precursor expressing TCF1 but not PLZF (127, 128). Despite evidence for such astonishing multiplicity of states of ILCs, what is less clear is whether these data are indicative of stable features of the cells or simply snapshots of transient states that emerge in many or all of the cells in a given population.

Recent methods allow for parallel measurement of single-cell transcriptomes and protein expression in addition to flow cytometry-based scRNA-seq techniques (129, 130). In the context of protein expression, time of flight mass cytometry followed by computational techniques has been used to dissect human ILCs in several tissues through evaluation of protein expression, based on a cytometry panel consisting of 38 transition element isotopes-labeled antibodies (131). Although this study contributes to unravel the complexity of ILCs between individuals and tissues, it also questions the existence of the prototypical ILC1 subset in human [as defined previously in Ref. (132)]. Beyond the technical details underlying the failure to reveal T-BET^+^ ILC1 in human tissues and the limitations of this approach [explained in Ref. (133)], the definition of human type 1 ILC populations remains a subject of debate, due to the absence of specific markers and the expression of molecules associated to T cell biology, including intracellular expression of CD3 and surface expression of CD5 (134, 135). Despite the increasing awareness of ILC heterogeneity, it will be important to clarify to what extent the degree of complexity defines truly distinct ILC subpopulations, with different functions, or whether reflects a range of activation states within a single population.

## Conclusion

Given that ILC regulomes are acquired independently of antigen receptor signals, a key unanswered issue is to understand how endogenous and exogenous factors function as drivers of chromatin organization. To what extent do endogenous TFs expressed during development contribute and how to exogenous and environmental factors including the microbiome, diet, and cytokines contribute to the development of the epigenomic landscape? What factors are common to all lymphocytes and which are subset or situationally specific? One prevailing view is that lineage-specifying TFs bind to thousands of places in the genome and have “pervasive” effects on high-order chromatin structure and accessibility of critical loci. We are beginning to understand these processes in B cells and T cells (136–138). Even for differentiated ILCs, it will be important to understand how acute activation does or does not continue to modify regulomes to drive gene expression. How do epigenomic modifications, chromatin looping, and transcription relate? Technical limitations related to the small numbers of ILCs limit our understanding of global three-dimensional chromatin structure at present; however, given the rate of advances in this field it seems unlikely that this will remain a barrier for long. Additionally, unraveling the signals present within the different bone marrow niches underlying acquisition of regulomes and functional specification will help to discriminate the signaling pathways involved.

The development of mouse models with conditional deletion of genes or cell types has helped to understand how similar ILCs and T cells are in terms of differentiation and function, and in which contexts they can play distinct roles. In human, primary immunodeficiency syndromes have revealed specific functions for NK cells in controlling viral infections (139, 140), while evidence for ILC redundancy has been recently provided (141). The appearance of distinct ILC subsets can precede that of B and T cells during vertebrate evolution (142), suggesting that prototypical effector programs have evolved along with the repertoire of effector lymphocytes. Of consequence, the redundant transcriptional/epigenetic regulation and, probably, functions of ILCs, innate-like, and adaptive T cells can be certainly seen as a key factor to improve the overall fitness of a species. Interestingly, the different effector programs appear to follow different routes of evolution. Indeed, type 2 cytokines seem to emerge after type 1 and type 3 cytokines, despite the early appearance of TFs belonging to the GATA family, implying an initial role in lymphoid development for GATA-3, above regulation of type 2 response (143, 144). Thus, one aspect to evaluate will be the degree of conservation relative to the SEs, spreading along cytokine loci, and defining cell identity, and the TFs underlying these important switches. The increased resolution and sensitivity of NGS technology has been providing a pivotal contribution to elucidate the mechanisms of epigenomic regulation underlying ILC development/effector functions and to identify REs distinctive of each ILC subset. The possibility of combining scRNA-seq and clustered regularly interspaced short palindromic repeats (CRISPR)-pooled screening will allow analysis of genomic perturbation on transcriptomes within the same cell (145, 146). Thus, this combination of genome-editing and single-cell techniques will enable elucidation of gene function and its loss-of-function phenotype at single-cell level, but also the functions of REs that contribute to identity, development and functions of lymphoid cells.

## Author Contributions

The final manuscript was a result of the joint efforts of all the authors.

## Conflict of Interest Statement

The authors declare that the research was conducted in the absence of any commercial or financial relationships that could be construed as a potential conflict of interest.
